# RT-dPCR in Mosquito Samples for ZIKV Detection: Effects of RNA Extraction and Reverse Transcription in Target Concentration

**DOI:** 10.3390/v12080827

**Published:** 2020-07-30

**Authors:** Paula Rodrigues de Almeida, Ana Karolina Antunes Eisen, Meriane Demoliner, Fernando Rosado Spilki

**Affiliations:** Laboratório de Saúde Única, Feevale Techpark, Universidade Feevale, Campo Bom 93700-000, Brazil; karolinaeisen@yahoo.com (A.K.A.E.); merianedemoliner@gmail.com (M.D.); fernandors@feevale.br (F.R.S.)

**Keywords:** RT-dPCR, ZIKV, quantification, mosquito

## Abstract

Zika virus (ZIKV) is an important arbovirus, responsible for recent outbreaks of Guillain Barré Syndrome and Congenital Zika Syndrome (CZS). After thousands of CZS cases, ZIKV is under constant surveillance in Brazil. Reliable and robust detection techniques are required to minimize the influence of host inhibitors from clinical samples and mosquito pool samples. Reverse transcription Digital Polymerase Chain Reaction (RT-dPCR) is a technique that allows the accurate quantification of DNA targets with high sensitivity, and it is usually less affected by inhibitors than RT-qPCR. This study aimed to assess the influence of mosquito tissue, RNA extraction and cDNA synthesis in ZIKV PCR detection. Samples containing 0, 3 and 10 mosquitoes were spiked with ZIKV MR766 and serially diluted prior to RNA extraction and RT-dPCR for ZIKV. Two reverse transcription protocols were tested. Assay sensitivity allowed the detection of 1.197 copies/µL. A higher correlation between dilution factor and target quantification was observed in 10 mosquito pool samples. The lower quantification in samples diluted without mosquitoes highlights the critical role of the reverse transcription step in RNA detection, since it could be attributed to reverse transcriptase variable performance in samples with low overall RNA concentration. The results in mosquito pools indicate that mosquito tissues do not inhibit ZIKV RT-dPCR, and the RT-dPCR technique has good sensitivity and robustness for ZIKV detection in mosquito pool samples regardless of mosquito tissue concentration.

## 1. Introduction

Arthropod transmitted flaviviruses are highly adapted to arthropod vectors at the species level, causing several degrees of disease outcomes. Mosquito borne flaviviruses derived from *Aedes* sp. have been transmitted throughout several continents over time and on several occasions. Flaviviruses, as most arboviruses, are established in sylvatic cycles involving animals and zoophilic arthropods as vectors. Spillover events involving forest dwelling mosquitoes infecting people can give rise to epidemics, with variable degrees of pathogenicity depending on the virus and the population affected [1]. Several strategies have been employed to investigate and recognize flaviviruses circulating in mosquitoes in sylvatic environments, and vector competence studies rely on viral detection in mosquito pools [2].

Zika Virus (ZIKV, *Flavivirus: Flaviviridae*) has become an important pathogen worldwide since the recent outbreaks that occurred in French Polynesia, Yap island and Northeastern Brazil, causing Guillain Barré Syndrome (GBS) and Congenital Zika Syndrome (CZS), respectively [3,4]. Since the CZS outbreak, ZIKV has been established in Brazil and is one of the arboviruses under constant surveillance, which is performed by detection in mosquito pools and clinical samples. The influence of host material in viral quantification and quality of viral nucleic acid purification could impair viral detection and quantification, consequently impairing surveillance systems. Reverse Transcription Digital Polymerase Chain Reaction (RT-dPCR) is a robust technique that allows for genomic copy number estimation with very narrow confidence intervals, target detection in very low concentrations and accurate analysis of variation in target number according to different treatments. The goal of this study was to assess the quantification of ZIKV genomic copies in mosquito pools containing 10, 3 and 0 mosquitoes to estimate the influence of host genetic material on target quantification, assay sensitivity and variation of precision rates according to treatment.

## 2. Materials and Methods

### 2.1. Sample Preparation

The experiment was designed to estimate the influence of different ZIKV concentrations in mosquito pool samples, therefore ZIKV MR766 was serially diluted in mosquito pools with diluent [2] modified with minimum essential medium (MEM) instead of PBS totaling three different raw sample categories: diluent containing 0 mosquitoes, 3 mosquitoes and 10 mosquitoes. MEM was chosen for dilution since it is routinely utilized in RNA extraction of field caught mosquitoes in our lab and for future viral isolation attempts. Mosquitoes from the pool belonged to the genus *Mansonia* sp. and were negative for *Flavivirus*. *Mansonia* sp. was utilized since this genus was readily available and does not transmit ZIKV, therefore the possibility of contamination from natural infection was excluded. ZIKV was diluted in from 10^−1^ to 10^−6^ in all treatments, prior to RNA extraction. 

Additionally, cDNA serial dilution of 4 samples with high viral concentration was performed in order to assess assay sensitivity of RT-dPCR using two distinct RT protocols.

### 2.2. RNA Extraction and cDNA Synthesis

RNA was extracted using TRIzol^®^ following the manufacturer’s protocol. Two protocols of reverse transcription (RT) were tested in all samples using SuperScript IV (Thermo Fisher Scientific^®^, Waltham, MA, U.S.) with random hexamers. Standard protocol was applied, where synthesis temperature was maintained at 50 °C for 10 min followed by 55 °C for 10 min, with inactivation at 80 °C; in the second protocol, a milder temperature gradient (MTG) was applied, with 42 °C for 20 min, 50 °C for 20 min, 55 °C for 20 min, 60 °C for 10 min and inactivation at 70 °C for 15 min. 

### 2.3. RT-dPCR

Two reactions of each cDNA sample were prepared for chip loading; in this way, there were two chips per sample. RT-dPCR reaction was performed in a total of 72 chips (6 dilutions, 3 treatments, 2 RT protocols, 2 reactions per sample). RT-dPCR experiments were conducted in a QuantStudio 3D^TM^ System (Applied Biosystems^TM^ Waltham, MA, U.S.), with the QuantStudio™ 3D Digital PCR Master Mix v2 dPCR mastermix and QuantStudio^TM^ 3D 20K v2 chips. Primers and probe targeting the ZIKV envelope, described by [3] to detect ZIKV with RT-qPCR, were used in RT-dPCR reactions. Cycling conditions for RT-dPCR differ from RT-qPCR assays and consist of 10 min at 96 °C, followed by 39 cycles of 30 s at 60 °C and 2 min at 98 °C, and a final step of 60 °C for 2 min followed by maintenance at 4 °C. 

### 2.4. Data Analysis

Initial data analysis was performed using the app dPCR AnalysisSuite^TM^ available at Thermo Fisher Connect^TM^ Dashboard. RT-dPCR results with precision values below 100% were used in the data analysis. The precision value is the percentage of variation of a value around a confidence interval [5]; precision values for low copy numbers can be near 100% and the confidence interval is less than 1 copy, usually, this parameter is higher in reactions with an excessive or very low target concentration. The quantitative linearity of the dilution series and the quantification results of the selected data in three different raw samples were assessed with a multiple regression analysis using Libre Office Calc spreadsheet functions.

## 3. Results

Data were selected based on the precision values obtained in the dPCR Analysis Suite^TM^ app, only precision values below 100% were used; therefore, some of the duplicates were excluded from the regression analysis. A data set with 43 quantification results of serially diluted ZIKV samples prior to RNA extraction was obtained, and the correlation between dilution factor and viral quantification obtained with different treatments is shown in Table 1.

**Figure 1:**
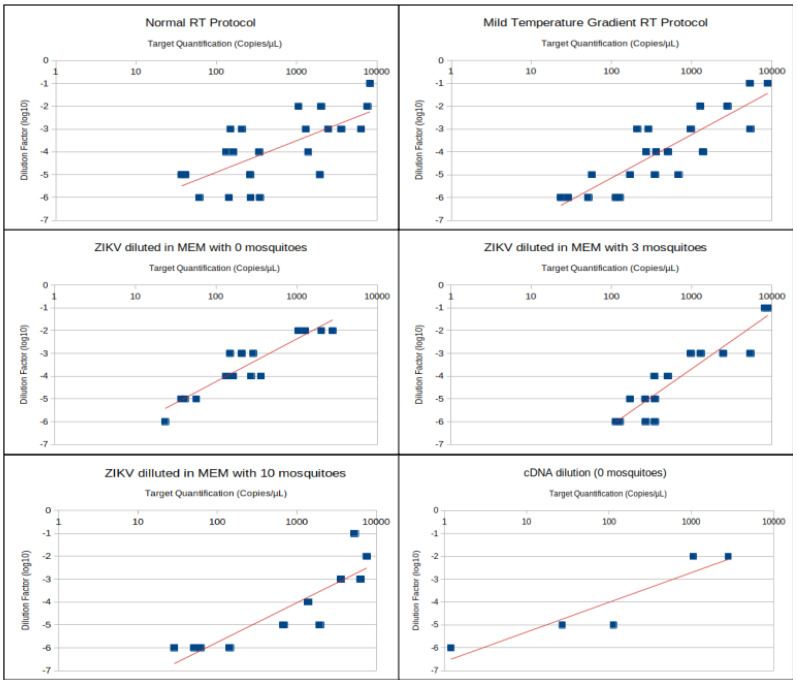
Dispersion charts with trend lines correlating dilution factors in log_10_ and viral quantification (logarithmic scale) for different samples and sample treatments. Results from milder temperature gradient (MTG) and normal reverse transcription (RT) protocols were pooled to create the panels of 0, 3 and 10 mosquitoes.

**Figure 2:**
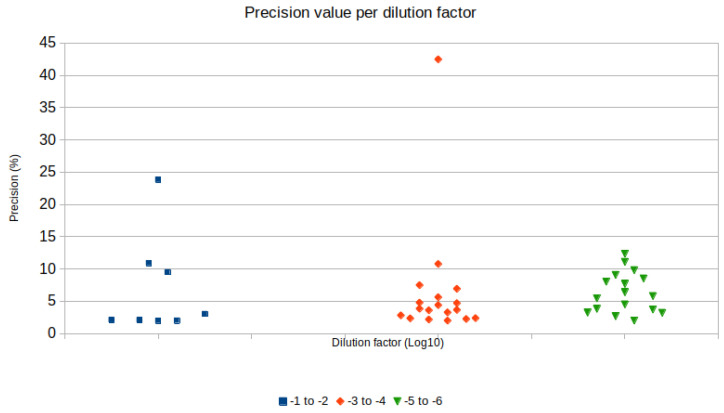
Distribution of precision values (y axis) according to the dilution factor grouped in 10^−1^ to 10^−2^, 10^−3^ to 10^−4^ and 10^−5^ to 10^−6^ (x axis).

Regression analysis was performed to analyze the linearity of sample quantification in different ZIKV concentrations in raw samples. Dilution in raw samples was preferred, as it mimics a realistic situation where unknown concentrations of a virus might be present in environmental samples, and sample processing in multiple steps is more likely to disrupt linearity due to cumulative pipetting errors. Regression charts according to sample treatment are displayed in Figure 1. Precision values were narrower and lower when the dilution factor was 10^−4^ or 10^−3^ (Figure 2).

Samples with ZIKV diluted in 10^−1^ and 10^−2^ resulted in excessively high viral concentrations, disturbing accurate target quantification. To adjust precision and resolve this issue, cDNA from 4 samples diluted with 0 mosquitoes was serially diluted, and 10^−4^ and 10^−8^ diluted samples were tested with RT-dPCR, and results below the established threshold of 100% for precision are shown in Table 2.

## 4. Discussion

RT-dPCR was able to detect a minimum of 1.197 copies of ZIKV per µL or 1197 genomic copies per mL. The primer probe assay used here was originally designed to detect ZIKV from patients through RT-qPCR and in that study, the Ct value for 900 genome copies per mL was 38 and for 1263 copies was 37 [3], Ct values that could be interpreted as inconclusive. In this study, we were able to conclusively detect the equivalent of 1197 copies per mL, equivalent to approximately 2–6 viral infectious particles per mL [3].

Serial dilutions from raw samples allowed us to detect 23.375 genomic copies per µL or 23,375 genomic copies per mL in a dilution factor of 10^−6^, which is equivalent to approximately 115 to 46 infectious particles per mL. Precision of this sample achieved 12%, meaning that target concentration in this sample was between 20 and 26 genomic copies per µL. Experimental ZIKV infection of mosquitoes shows that usually a ZIKV positive mosquito has approximately 10⁴ genomic copies [6]. The sensitivity of RT-dPCR allows for ZIKV detection with similar sensitivity (≃2.8 × 10⁴) in a pool of 10 mosquitoes, which could represent 2.8 x 10^3^ genomic copies per mosquito. Moreover, mosquito pools of 10 individuals did not inhibit RT-dPCR reactions, in fact the quantification of ZIKV diluted with 10 mosquitoes was apparently better correlated to the dilution factor than the other two treatments, reaching a correlation near to 0.9. Nevertheless, more samples presented precision values above the threshold in this treatment and were discarded; consequently, there were less samples included in the calculation of correlation when comparing to dilutions in 0 and 3 mosquitoes (Table 1).

A lower correlation between dilution factor and copies/µL was observed in MEM diluted samples. The TRIzol^TM^ RNA extraction method results in high yields and acceptable purity; however, it has been shown that small RNAs depend upon carrier molecules to be efficiently extracted by this method, and starting with a higher concentration of large RNA molecules influences extraction [7]. It is difficult to draw any conclusions of such methodology of TRIzol^TM^ RNA extraction interference in our samples, since viral stock always has host cell RNA along with viral RNA. Moreover, reverse transcriptase variability is usually higher with a low RNA concentration [8], and in zero mosquito pools, overall RNA concentration was lower than in mosquito pools, since the spiked ZIKV MR766 RNA and host cell RNA were the only RNA in these samples, while in mosquito pools a substantial amount of mosquito RNA was added to the sample.

Serial dilution is a fundamental practice to achieve good precision in RT-dPCR experiments [5]. In this study, the serial dilution of cDNA from MEM diluted samples with excessive target copy numbers that could not be quantified in the first RT-dPCR reaction allowed accurate target quantification. Ten-fold serial dilutions from cDNA samples with ZIKV diluted in 10^−1^ and 10^−2^ were performed, and a 10^−4^ dilution factor resulted in a precision of 5%, quantifying 112 copies (ranging from 106.65 to 119.17 copies/µL) in the MGT RT protocol and a precision of 11% with 25 copies (ranging from 24.105 to 29.927 copies/µL) in the RT normal protocol. Precision is a parameter calculated in the Analysis Suite^TM^ app, and represents the percentage of variation of the confidence interval around the copy number estimation calculated based on positive partitions in the chip with the assay. Thus, quantification accuracy is inversely related to the precision value of an RT-dPCR assay [5]. The ideal partition occupancy for accurate quantification requires approximately 20% of negative partitions in an RT-dPCR assay with the platform used in this study [5]. Dilution factors of 10^−3^ and 10^−4^ in raw samples resulted in a target concentration that met this requirement, therefore presented better precision values; most of the precision values for these samples were below 5% (Figure 2).

RNA viruses impose difficulties in molecular detection and transcriptomic analyses due to RNA instability when compared to DNA and the additional RT step required during sample processing [9]. Reverse transcription is a highly variable step and multiple options and recommendations are often controversial and inconsistent [9,10]. The improvement of PCR amplification efficiency with gradually increasing RT temperature has been shown for human immunodeficiency virus (HIV). During the RT step, oligo mismatches can impair PCR amplification and target detection [11]. The sample processing protocol with 10 mosquitoes and temperature ramp RT presented the best correlation (r = 0.98) between target copies per µL and ZIKV dilution factor in the raw sample. Hence, this protocol seems to be more reliable for viral quantification in mosquito pool samples. Nonetheless, there were only a few samples included in the r calculation for these samples, since many of them presented precision above the threshold. The MTG RT presented a better correlation between dilution factor and copies/µL than normal protocol, moreover, this protocol allowed the detection of 1.19 copies/µL at a dilution factor of 10^−6^ with a precision of 72.2% (ranging from 0.695 to 2.06 copies/µL). 

The use of *Mansonia* sp. mosquitoes is a limiting factor to this study. Factors such as enzymes, proteins and host RNA could impair assay sensitivity in different levels for *Mansonia* sp. and *Aedes* sp. mosquitoes. Since we did not use *Aedes* sp. mosquitoes in the experiments and *Mansonia* sp. is far less known than *Aedes* sp. [12], unknown factors could interfere with RT, RNA extraction and RT-dPCR. The use of *Aedes* sp. pools for these experiments would mimic a more realistic situation, yet mosquito rearing conditions would be necessary to discard the possibility of ZIKV natural infection.

## 5. Conclusions

This study has demonstrated that RT-dPCR is a sensitive and precise detection technique for ZIKV quantification in mosquito pool samples. Samples with 10 mosquitoes did not inhibit RT-dPCR detection of ZIKV; conversely, the correlation between target quantification and dilution factor was higher with 10 than with 0 mosquitoes. The only limiting factor to RT-dPCR is the same as for other amplification based detection techniques targeting RNA: reverse transcription variability. Nonetheless, due to its robustness and capability of achieving very low error rates in quantification, RT-dPCR can be utilized in RT optimization for several purposes such as viral detection and quantification, and virome or transcriptome library preparation. The dilution of cDNA allowed the achievement of ideal precision values with a confidence interval of very few copy numbers; this method is valuable for the construction of quantification curves to be used in RT-qPCR. This is especially useful for viruses that are difficult to grow in cell culture and for pathogens requiring high biosafety levels to be manipulated.

## Figures and Tables

**Table 1:** Correlation between dilution factor and target quantification according to treatment.

Treatment	Number of Samples Used for r Calculation	Correlation (r)
0 mosquitoes (normal RT)	8	0.765
0 mosquitoes (MTG RT)	8	0.702
3 mosquitoes (normal RT)	7	0.845
3 mosquitoes (MTG RT)	8	0.86
10 mosquitoes (normal RT)	7	0.943
10 mosquitoes (MTG RT)	5	0.988
0 mosquitoes (total)	16	0.713
3 mosquitoes (total)	15	0.848
10 mosquitoes (total)	12	0.89
Normal RT (total)	22	0.65
MTG RT (total)	21	0.726

MTG - mild temperature gradient; RT - reverse transcription.

**Table 2:** Digital Polymerase Chain Reaction (RT-dPCR) results after cDNA dilution of 0 mosquito samples with high viral concentrations.

Sample/RT Protocol	Copies/µL	CI	Precision	Sample Dilution	cDNA Dilution
1:100 MTG	2794.8	2737.1–2853.7	2.11%	10^−2^	pure
1:100 2T	1052.1	1031.2–1073.4	2.02%	10^−2^	pure
1:10 MTG	112.74	106.65–119.17	5.71%	10^−1^	10^−4^
1:10 2T RT	26.858	24.105–29.927	11.42%	10^−1^	10^−4^
1:100 MTG	1.197	0.695–2.061	72.22%	10^−2^	10^−4^

2 T—two temperature RT protocol; MTG—mild temperature gradient RT protocol; CI—confidence interval.

## References

[1] Moureau G., Cook S., Lemey P., Nougairede A., Forrester N., Khasnatinov M., Charrel R.N., Firth A.E., Gould E.A., Lamballerie X. (2015). New insights into Flavivirus evolution, taxonomy and biogeographic history, extended by analysis of canonical and alternative coding sequences. PLoS ONE.

[2] Fauver J.R., Grubaugh N.D., Krajacich B.J., Weger-Lucarelli J., Lakin S.M., Fakoli L.S., Bolay F.K., Diclaro J.W., Dabiré K.R., Foy B.D. (2015). West African Anopheles gambiae mosquitoes harbor a taxonomically diverse virome including new insect-specific flaviviruses, mononegaviruses, and totiviruses. Virology.

[3] Lanciotti R.S., Kosoy O.L., Laven J.J., Velez J.O., Lambert A.L., Johnson A.J., Stanfield S.M., Duffy M.R. (2008). Genetic and serologic properties of Zika virus associated with an epidemic, Yap State, Micronesia, 2007. Emerg. Inf. Dis..

[4] Mlakar J., Korva M., Tul N., Popovic M., Pljsak-Prijatelj M., Mraz J., Kolenk M., Rus K.R., Vipotnik T.V., Vodusek V.F. (2016). Zika virus associated with microcephaly. N. Eng. J. Med..

[5] Majumdar N., Wessel T., Marks J. (2015). Digital pcr modeling for maximal sensitivity, dynamic range and measurement precision. PLoS ONE.

[6] Du S., Liu Y., Liu J., Zhao J., Champagne C., Tong L., Zhang R., Zhang F., Qin C., Ma P. (2019). Aedes mosquitoes acquire and transmit Zika virus by breeding in contaminated aquatic environments. Nat. Commun..

[7] Kim Y.K., Yeo J., Kim B., Ha M., Kim V.N. (2012). Short structured RNAs with low GC content are selectively lost during extraction from a small number of cells. Mol. Cell..

[8] Bustin S., Dhillon H.S., Kirvell S., Greenwood C., Parker M., Shipley G.L., Nolan T. (2015). Variability of the reverse transcription step: Practical implications. Clin. Chem..

[9] Minshal N., Git A. (2020). Enzyme- and gene-specific biases in reverse transcription of RNA raise concerns for evaluating gene expression. Sci. Rep..

[10] Schwaber J., Andersen S., Nielsen L. (2019). Shedding light: The importance of reverse transcription efficiency standards in data interpretation. Biomol. Detect. Quantif..

[11] Christopherson C., John Sninsky J., Kwok S. (1997). The Effects of Internal Primer-Template Mismatches on RT-PCR: HIV-1 Model Studies. Nucleic Acids Res..

[12] Beerntsen B.T., James A.A., Christensen B.M. (2000). Genetics of Mosquito Vector Competence. Microbiol. Mol. Biol. Rev..

